# X‐ray multimeter performance under calibration laboratory conditions

**DOI:** 10.1002/mp.18106

**Published:** 2025-09-03

**Authors:** John T. Stasko, Wesley S. Culberson

**Affiliations:** ^1^ Department of Medical Physics School of Medicine and Public Health University of Wisconsin‐Madison Madison Wisconsin USA

**Keywords:** air‐kerma rate, half‐value layer, quality assurance, X‐ray multimeters

## Abstract

**Background:**

Estimating dose delivered to patients for a typical mammographic or radiologic examination requires accurate knowledge of several beam quantities. X‐ray multimeters (XMMs) are compact, solid‐state semiconductor dosimeters that have become common for conventional QA measurements due to their ease of use.

**Purpose:**

In this investigation, the performance of two XMMs in low‐energy x‐ray calibration beams was assessed, and the stability of the manufacturer's calibration over time was evaluated.

**Methods:**

An RTI Piranha and Radcal Accu‐Gold+ with AGMS‐DM+ sensor were used to measure air‐kerma rates and half‐value layers of UW‐MO and UW‐M series calibration beams. Measurement results were compared to reference values collected with standard instruments. The same measurements were repeated every 3–5 months for 2.5 years to evaluate whether the XMMs’ energy response changes over time.

**Results:**

All air‐kerma rate measurements and nearly all half‐value layer measurements were within tolerance of the reference measurements. Both XMMs were satisfactorily stable over the course of the study, with all measured air‐kerma rates within a 2% range relative to the reference for a specific beam. However, some drift in response was observed, particularly for the RTI Piranha.

**Conclusions:**

Both XMMs met the performance standards provided by their respective manufacturers. An additional calibration would result in increased measurement accuracy for some beam series. One limitation of this study is that the detectors were not subject to more rigorous clinical conditions.

## 1 INTRODUCTION

Low‐energy x‐ray beams are used in many imaging tasks, such as mammography, fluoroscopy, and computed tomography. However, these imaging exams expose patients to ionizing radiation, so it is vital to consider the impact of absorbed dose on the patient.

Estimating dose delivered to patients requires accurate knowledge of several quantities of the beam, such as half‐value layer (HVL) and air‐kerma rate (AKR).^1^ Measurements of these quantities are regularly collected as part of standard QA processes. In order to measure these characteristics of the beam accurately, calibrated dosimeters are required.^2^ These dosimeters are often ionization chambers. Some solid‐state semiconductor dosimeter systems, referred to as x‐ray multimeters (XMMs), have come into use as an easy‐to‐operate alternative.

XMMs estimate various beam quantities, such as tube potential, AKR, and HVL, in a single exposure, with readout performed via dedicated software. The user must select the most appropriate calibration to use before measurements. However, no standard process for calibrating these devices currently exists, and all calibrations are done by the device's manufacturer. Additionally, it is unclear how conditions that are different from how the XMM was calibrated affect its response.

The International Electrotechnical Commission (IEC) 61674 defines a limit of ±5% on variation in response due to energy differences within typical mammographic or computed tomography conditions.^3^ Several researchers have evaluated the performance of various XMMs in mammographic clinical^4^, ^5^, ^6^ and calibration^6^, ^7^, ^8^, ^9^ conditions. Similar work has been done for typical radiography and fluoroscopy conditions.^10^, ^11^, ^12^ However, a number of these studies do not reflect current versions of either software or the XMMs themselves.

This study is the first to examine the performance of XMMs in mammography and radiology calibration beams in the United States. The accuracy of AKR and HVL measurements was evaluated relative to the corresponding reference standards. We also investigated the consistency of AKR measurements over time to determine whether manufacturer‐suggested calibration periods were reasonable.

## 2 MATERIALS AND METHODS

### 2.1 Calibration beams

At the University of Wisconsin Accredited Dosimetry Calibration Laboratory (UWADCL), there are several x‐ray beam series used for calibration of diagnostic imaging dosimeters. The series used in this project were the UW‐MO, Mo‐anode, Mo‐filter beams for mammography calibrations, and the UW‐M, W‐anode, moderately filtered (Al or Al and Cu filtration) beams for CT and fluoroscopy calibrations. These beams have greater filtration than the other UW radiographic beam series, the UW‐L series or lightly filtered series. Beam quantities for each beam used in this work are provided in Table 1. UW‐MO series beams were produced using a Lohmann (Leverkusen, Germany), model 50/40Mo, x‐ray tube with a 30° anode angle, 6×8 mm^2^ focal spot, and 1 mm Be inherent filtration. The UW‐M series beams were produced using a Comet (Shelton, CT), model MXR‐320/26, x‐ray tube with a 20° anode angle, 5.5 × 5.5 mm^2^ focal spot, and 3 mm Be inherent filtration.

**TABLE 1:** Description of parameters and quantities for all beams used in this project.

Beam	Filter	Nominal tube potential (kV)	Tube current (mA)	AKR at 1 m from source (mGy)	HVL (mm Al)
UW 23‐MO	0.032 mm Mo	23	20	0.568	0.280
UW 25‐MO	0.032 mm Mo	25	20	0.733	0.305
UW 28‐MO	0.032 mm Mo	28	20	0.992	0.336
UW 30‐MO	0.032 mm Mo	30	20	1.243	0.359
UW 35‐MO	0.032 mm Mo	35	20	1.795	0.390
UW 60‐M	1.66 mm Al	60	25	1.758	1.68
UW 80‐M	3.09 mm Al	80	25	1.737	3.07
UW 100‐M	5.21 mm Al	100	25	1.746	5.10
UW 120‐M	2.21 mm Al + 0.13 mm Cu	120	25	2.417	6.78
UW 150‐M	2.49 mm Al + 0.31 mm Cu	150	20	2.037	10.30

Abbreviations: AKR, air‐kerma rate; HVLs, half‐value layers.

The x‐ray tubes in this study share one collimator housing with separate primary collimators. Each x‐ray tube has an independent shutter that can be opened or closed regardless of whether the beam is on or off. The collimators and monitor chambers for each tube are fixed in place, while the individual filter packs can be removed to change the beam quality.

While tungsten anodes have become common for mammographic x‐ray tubes, the UWADCL does not currently have any available tungsten‐anode mammography calibration beams. Therefore, we were unable to evaluate the performance of the XMMs in those beams.

### 2.2 X‐ray multimeters

The two x‐ray multimeters investigated in this project were an RTI (Mölndal, Sweden) Piranha and a Radcal (Monrovia, CA) Accu‐Gold+ with AGMS‐DM+ sensor. These devices were purchased in March 2022, and the manufacturers were not involved in this study. The XMMs are capable of determining AKR, tube potential, HVL, and other quantities for mammography and other diagnostic radiology procedures. Depending on the measurement conditions, the user must select the closest calibration option within the software that is appropriate for their purposes.

RTI suggests a 2‐year calibration period, while Radcal suggests a 1‐year calibration period for their device. The RTI Piranha was calibrated on February 25, 2022, and the Radcal AGMS‐DM+ was calibrated on March 24, 2022. Neither device was sent for recalibration over the course of the study. Manufacturer‐specified confidence limits on measurements are provided in Table 2.

**TABLE 2:** Manufacturer‐specified confidence limits on measurements with the XMMs.

Device	AKR	HVL
RTI Piranha^15^	Radiography: ±5% or ±7 nGy/s Mammography: ±5% or ±12 nGy/s	Radiography: ±10% or ±0.2 mm Mammography: ±10%
Radcal AGMS‐DM+^14^	±5%	±5%

*Note*: When multiple values are listed, the larger of the two is the confidence limit.

Abbreviations: AKR, air‐kerma rate; HVLs, half‐value layers; XMM, X‐ray multimeters.

### 2.3 Measurement procedure

Measurements of AKRs and HVLs were collected with both XMMs and compared to values from reference standards. The reference standard for measuring UW‐MO series beams’ AKRs was a Keithley (Beaverton, OR) 96035 parallel‐plate ion chamber, S/N: 18691. The reference standard for measuring UW‐M series beams’ AKRs was an Exradin (Standard Imaging Inc., Middleton, WI) A3 spherical ion chamber, S/N: XR022483. Both of these chambers were calibrated at the National Institute of Standards and Technology.

The reference standard for HVL measurements of the UW‐M or UW‐MO beams was the Attix free‐air chamber (FAC). This chamber was designed for absolute measurements of AKRs for beams with maximum photon energy below 50 keV. For HVL measurements, it is treated as a large, wall‐less ionization chamber. The lack of a wall is important for low‐energy beams, where the wall or entrance window of another chamber may harden the beam, and thus, skew the HVL measurement to be greater than its actual value.

HVL measurements with the Attix FAC followed the standard procedure for measuring HVLs.^13^ The aperture of the Attix FAC was placed 100 cm from the source, and charge measurements were collected over either 30 or 60 s. Then, pure Al (4N or higher) attenuating sheets were placed 50 cm from the source. Three different total thicknesses of Al were used, such that they reduced the measured charge to near 50% of its original value. The ratios of the measured charges to the charge without any Al attenuators were plotted versus the thickness of the Al attenuators. Using a logarithmic scale for the vertical axis allowed a line of best fit to be applied to the data. The thickness of Al that corresponds to the point on the best‐fit line where the charge ratio is equal to 0.50 was taken as the HVL of that beam.

**FIGURE 1:**
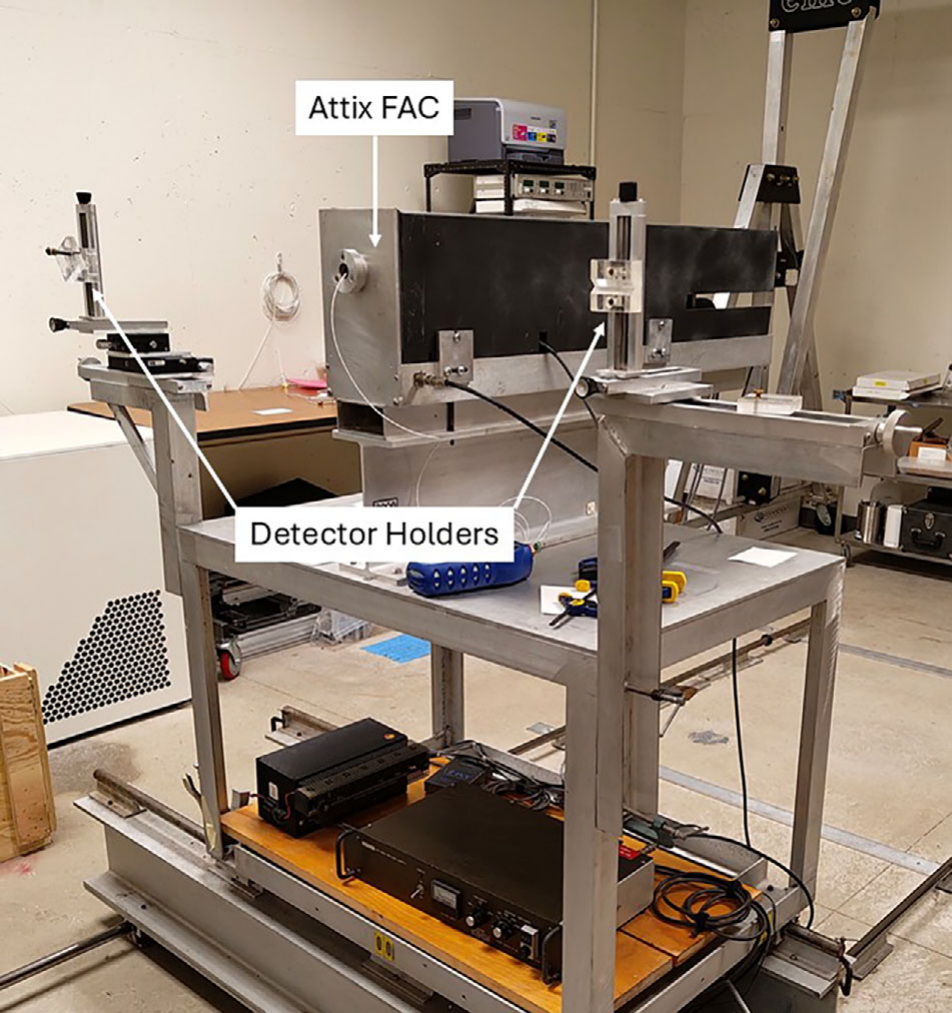
Photograph of the alignment cart. There are two detector holders, one for each x‐ray tube, and the Attix free‐air chamber.

All measurements in this project were collected using a typical calibration geometry: a 10×10 cm^2^ field size at 100 cm from the x‐ray source. XMMs and reference ion chambers were placed along the beam axis with the aid of lasers and a custom alignment cart, pictured in Figure 1. The cart is mounted on rails, allowing for simple gross adjustments along two axes. The alignment stages on either side of the cart allowed for precise positioning in either the Mo‐anode or W‐anode x‐ray beams. The alignment stages also provided a stable way to suspend the dosimeters in air without substantial sources of scatter around them.

Measurements with the XMMs were acquired using the corresponding manufacturer‐provided software: Accu‐Gold 2, version 2.55, batch 1 for the Radcal system and Ocean Next™, version 1.0.2.27272 for the RTI Piranha. For each measurement, the most applicable anode‐filter calibration was selected. The calibrations and their specified measurement conditions are listed in Table 3. Measurements with the RTI Piranha were collected using the “Compression paddle not used” option. For measurements with the Radcal AGMS‐DM+, it was discovered that the 2.2 mm polycarbonate sheath meant to mimic the scatter and attenuation of a compression paddle greatly decreased measurement accuracy, so the sheath was removed for all measurements reported in this study.

**TABLE 3:** Calibration options chosen within the relevant software for measurements in the UW‐MO or UW‐M series beams.

Device	UW‐MO series	UW‐M series
RTI Piranha^15^	Mammography Mo/30 µm Mo calibration: Tube Potential: 18–49 kV AKR: 25 nGy/s–530 mGy/s HVL: 0.19–0.47 mm Al	Radiography calibration: Tube Potential: 35–160 kV AKR: 15 nGy/s–320 mGy/s HVL: 1.2–14 mm Al
Radcal AGMS‐DM+^14^	Mammography Mo/Mo General calibration: Tube Potential: 21–49 kV AKR: 40 nGy/s–165 mGy/s HVL: 0.21–0.50 mm Al	Rad/Fluoro/Dental W/Al General calibration: Tube Potential: 50–160 kV AKR: 40 nGy/s–165 mGy/s HVL: 1.3–13.5 mm Al

Five measurements of at least 20 s were collected with each XMM for each x‐ray beam. The beam shutter was closed while the x‐ray beam was turned on. The shutter was opened to begin a given measurement after at least 10 s had passed since beam‐on and set to close after 20 s had elapsed. The value reported by the XMM was the average of the given quantities over the measurement duration. Early measurements were performed over more than 20 s, but it was deemed unnecessary to measure for longer, as the average results did not change from a longer measurement duration to the shorter measurement duration. Average measured AKRs were compared to rates measured by the standard parallel‐plate chambers on the same day. Average measured HVLs were compared to reference values measured in 2022, for data collected in 2022. Measured HVLs collected after the beginning of 2023 were compared to reference HVLs measured in March 2023. This change was made as the monitor chamber and filters for these beams were updated then.

### 2.4 Longitudinal study

In order to study the performance of both XMMs over time, the same AKR measurements were repeated approximately every 3 to 5 months. This was done to evaluate how each XMM's response might vary over time, both within and beyond the manufacturer's suggested calibration period. We wished to test whether the measurement results became less accurate over time or if there was a clear drift in the measurements over time.

Measurement results were collected over nearly 30 months. To determine if a trend exists in the measured data for any beam, a *t*‐test for linear regression was performed on the linear fit of the percent difference from the reference standard versus the measurement month for each of the 10 beams in this study. These tests were carried out in *R* (www.r‐project.org). The null hypothesis for these tests is that no linear trend appears in the data. For each beam's linear fit, the corresponding summary provided the slope of the fit, the standard error of that value, and the probability of having that slope given the true relationship between the dependent and independent variables has no trend. If the probability was sufficiently small, we could conclude that there is significant evidence to reject the null hypothesis and that a linear trend does exist in the time data.

Although HVL measurements were acquired at the same time as AKR measurements for all beams, the measured values showed no change over the entire study for the majority of the investigated beams. Any observed changes were on the order of single microns of Al. Therefore, the HVL measurement results were considered stable, and no statistical analysis was performed on that data.

## 3 RESULTS

### 3.1 AKR measurements

**FIGURE 2:**
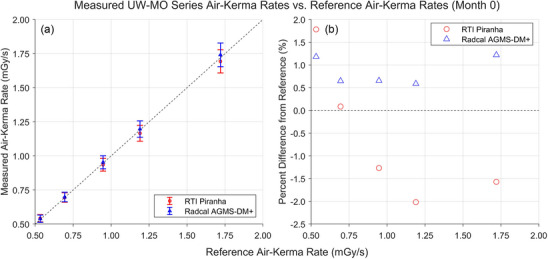
Results of AKR measurements for the UW‐MO beams completed in September 2022. (a) Comparison between the AKRs measured by the XMMs and the AKRs measured by the reference standard. (b) Percent difference between the AKRs measured by the XMMs and the reference AKRs. AKR, air‐kerma rate; XMM, X‐ray multimeters.

**FIGURE 3:**
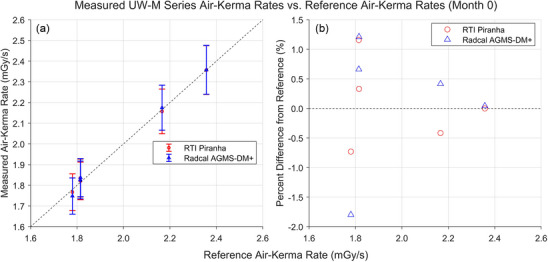
Results of AKR measurements for the UW‐M beams completed in August 2022. (a) Comparison between the AKRs measured by the XMMs and the AKRs measured by the reference standard. (b) Percent difference between the AKRs measured by the XMMs and the reference AKRs. AKR, air‐kerma rate; XMM, X‐ray multimeters.

Figures 2 and 3 show the results of AKR measurements performed in August or September 2022, month 0 for either the UW‐M or UW‐MO beam measurements, respectively. All measured AKRs fell between ‐2.0% and +1.8% of the reference values. The error bars in the following figures are the manufacturer‐specified confidence limits.

**FIGURE 4:**
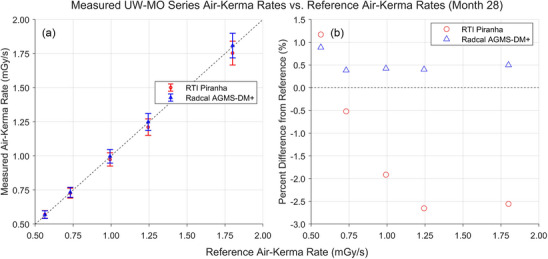
Results of AKR measurements for the UW‐MO beams completed in January 2025, 28 months after the start of the study. (a) Comparison between the AKRs measured by the XMMs and the AKRs measured by the reference standard. (b) Percent difference between the AKRs measured by the XMMs and the reference AKRs. AKR, air‐kerma rate; XMM, X‐ray multimeters.

**FIGURE 5:**
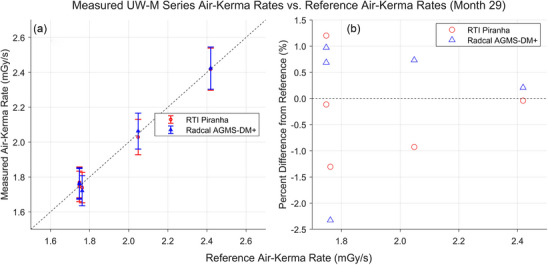
Results of AKR measurements for the UW‐M beams completed in January 2025, 29 months after the start of the study. (a) Comparison between the AKRs measured by the XMMs and the AKRs measured by the reference standard. (b) Percent difference between the AKRs measured by the XMMs and the reference AKRs. AKR, air‐kerma rate; XMM, X‐ray multimeters.

Results of the most recent AKR measurements with the XMMs are shown in Figures 4 and 5. These measurements were performed 28 or 29 months after the start of the measurement series, with both XMMs beyond the manufacturers’ suggested calibration dates. All measured AKRs fell between −2.7% and +1.2% of the reference values.

### 3.2 HVL measurements

**FIGURE 6:**
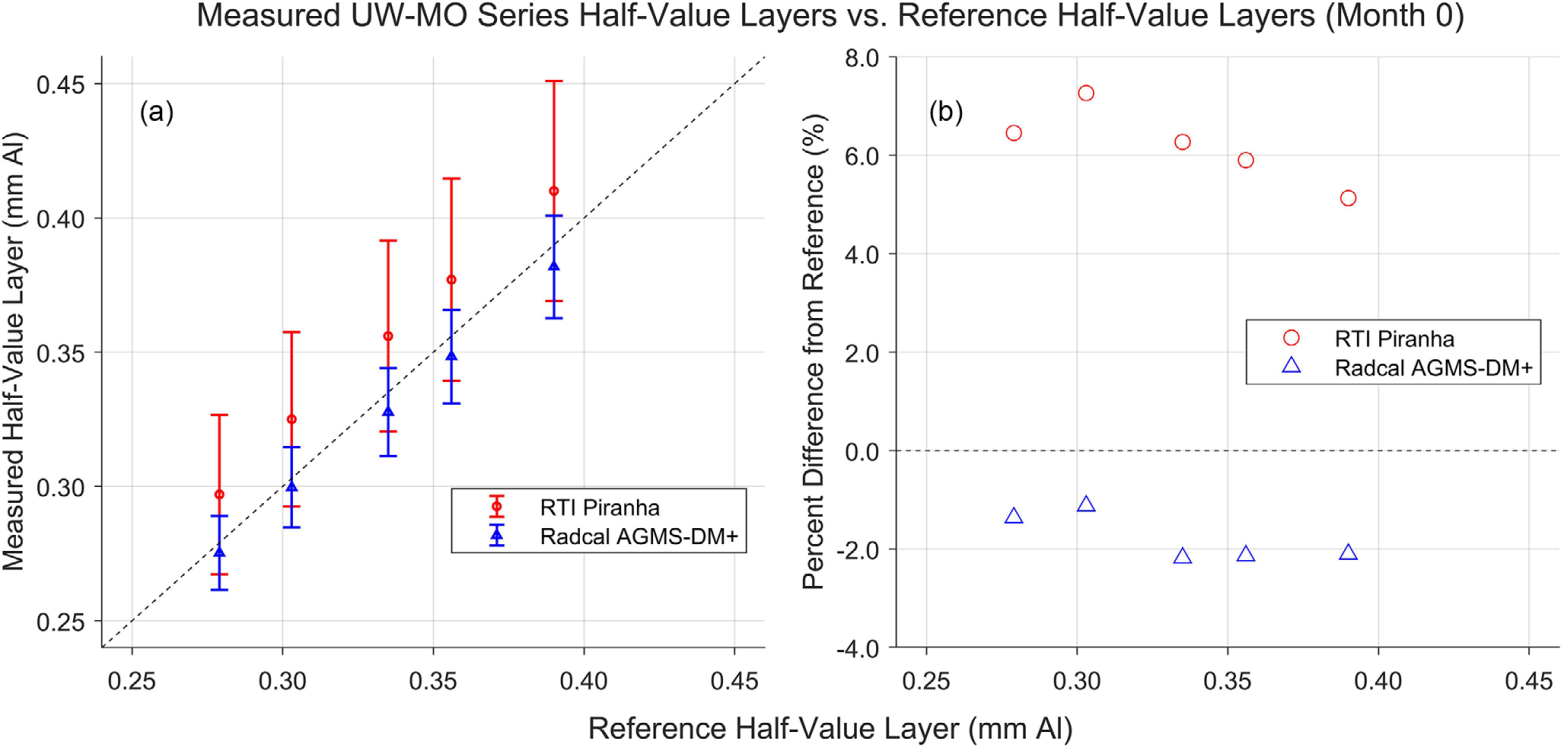
Results of HVL measurements for the UW‐MO beams completed in September 2022. (a) Comparison between the HVLs measured by the XMMs and the HVLs measured by the reference standard. (b) Percent difference between the HVLs measured by the XMMs and the reference HVLs. HVLs, half‐value layers; XMM, X‐ray multimeters.

**FIGURE 7:**
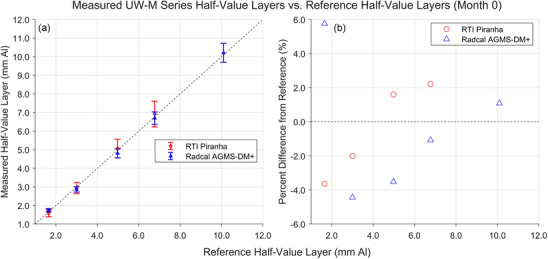
Results of HVL measurements for the UW‐M beams completed in August 2022. a) Comparison between the HVLs measured by the XMMs and the HVLs measured by the reference standard. b) Percent difference between the HVLs measured by the XMMs and the reference HVLs. HVLs, half‐value layers; XMM, X‐ray multimeters.

Figures 6 and 7 show the results of HVL measurements performed in August or September 2022, month 0 for the UW‐M or UW‐MO series measurements, respectively. All measured HVLs fell between −4.4% and +7.3% of the reference values. The RTI Piranha was unable to measure the HVL of the UW 150‐M beam.

**FIGURE 8:**
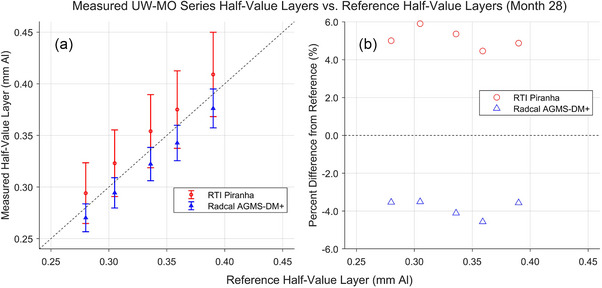
Results of HVL measurements for the UW‐MO beams completed in January 2025, 28 months after the start of the study. (a) Comparison between the HVLs measured by the XMMs and the HVLs measured by the reference standard. (b) Percent difference between the HVLs measured by the XMMs and the reference HVLs. HVLs, half‐value layers; XMM, X‐ray multimeters.

**FIGURE 9:**
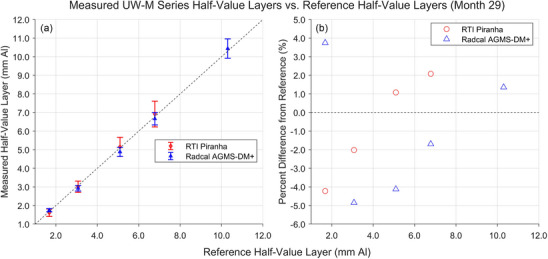
Results of HVL measurements for the UW‐M beams completed in January 2025, 29 months after the start of the study. a) Comparison between the HVLs measured by the XMMs and the HVLs measured by the reference standard. b) Percent difference between the HVLs measured by the XMMs and the reference HVLs. HVLs, half‐value layers; XMM, X‐ray multimeters.

Results of the most recent HVL measurements with the XMMs are shown in Figures 8 and 9. Again, these measurements were performed 28 or 29 months after the start of the study. All measured HVLs fell between −4.9% and +5.9% of the reference values. Again, the Piranha did not provide a reading for the UW 150‐M beam.

### 3.3 Longitudinal study

**FIGURE 10:**
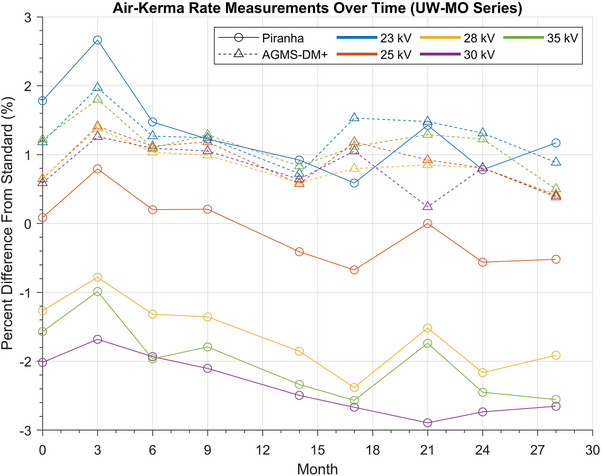
AKR measurement results of UW‐MO beams over time. The line marker and style indicate the XMM that was used to acquire the measurements, and the color of the line indicates the nominal tube potential of that beam. X‐ray multimeters.

**FIGURE 11:**
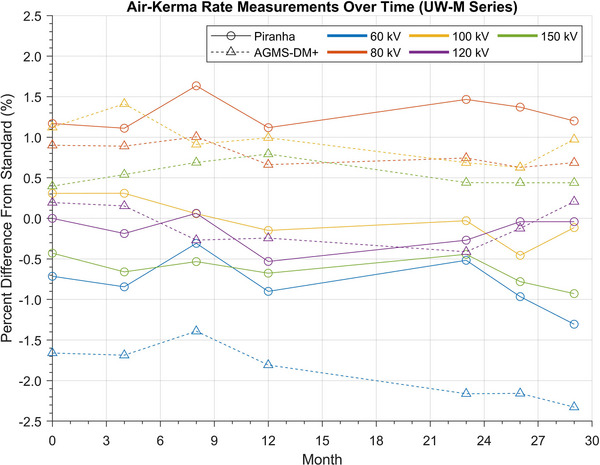
AKR measurement results of UW‐M beams over time. The line marker and style indicate the XMM that was used to acquire the measurements, and the color of the line indicates the nominal tube potential of that beam. AKR, air‐kerma rate; XMM, X‐ray multimeters.

Results of the longitudinal study are shown in Figures 10 and 11. The percent difference of the measurement from the reference standard is plotted versus the number of months since the beginning of the study. Each data point is within 5 months of the previous data point, other than months 13–22 of the UW‐M series measurements, where no data was acquired.

Overall, most of the measured AKRs appear consistent over the course of the study, with some potential drift in the XMMs’ responses relative to the standard. The maximum change relative to the standard for the UW‐MO beams was the RTI Piranha's measured AKR for the UW 35‐MO beam, where the result changed from −1.6% relative to the standard to −2.6% relative to the standard. The RTI Piranha also showed the greatest range of measured values relative to the standard for a single beam, as the percent difference from the standard value for the UW 23‐MO beam varied from 0.6% to 2.7% over the course of the study. The UW‐M series measurements were more stable. The largest change relative to the standard was the Radcal AGMS‐DM+ measurements for the UW 60‐M beam, which changed from −1.7% relative to the standard in August 2022 to −2.3% relative to the standard in January 2025. The largest range of differences to the standard was for the Piranha measurements of the UW60‐M beam, which varied between 0.3% and −1.3% relative to the reference standard over the course of the study.

A linear model was fit to every series of measurements in the longitudinal study. A *t*‐test for linear regression was performed on every linear fit to determine if the trend line was significantly different from no trend. Resulting *p*‐values from the statistical analyses are provided in Table 4.

**TABLE 4:** *P*‐values from the *t*‐tests for linear regression on all beams in the long‐term AKR study.

Beam	RTI Piranha	Radcal AGMS‐DM+
UW 23‐MO	0.063	0.407
UW 25‐MO	0.021^*^	0.201
UW 28‐MO	0.022^*^	0.127
UW 30‐MO	0.001^**^	0.140
UW 35‐MO	0.023^*^	0.104
UW 60‐M	0.279	0.007^**^
UW 80‐M	0.642	0.047^*^
UW 100‐M	0.030^*^	0.075
UW 120‐M	0.953	0.622
UW 150‐M	0.149	0.529

* : Significant at 5% level

** : Significant at 1% level.

Abbreviation: AKR, air‐kerma rate.

## 4 DISCUSSION

### 4.1 AKR measurements

AKR measurements with both XMMs were within manufacturer‐specified tolerances of the reference‐standard measured AKRs for both the UW‐MO and UW‐M series beams. The largest percent difference from the reference value was −2.7%. This is well within the response variation criteria of ±5% from IEC 61674.^3^

For the UW‐MO beams, the Radcal AGMS‐DM+ was the more accurate and precise dosimeter. However, XMM consistently overestimated the AKR, indicating that an additional calibration factor would improve the accuracy of measurements acquired with the AGMS‐DM+ in the UW‐MO calibration beams. In contrast, the RTI Piranha overresponded to the lowest tube potential UW‐MO beams, then underresponded to the higher tube potential UW‐MO beams. An energy‐dependent calibration might improve the accuracy of these measurements.

Both devices performed similarly in the UW‐M beams. The measurements that were most different from the reference AKRs were for the UW 60‐M beam. This may be because the Al filter selected for the UW 60‐M beam was below the suggested operational range for the AGMS‐DM+ (2–40 mm total filtration)^14^, so the device may not perform as consistently in that beam.

Some strange behavior was observed for measurements with the RTI Piranha. Sometimes, the reported value for the AKR, as read directly off of the software, was markedly different from previous measurements. However, based on the time of the irradiation and the total air kerma collected by the Piranha, the reported average AKR was incorrect. The true value was consistent with the expected values of the AKR. This error appeared randomly, with a frequency of about once in every 50 measurements.

### 4.2 HVL measurements

HVL measurements with the XMMs were generally less accurate than the AKR measurements. This was not unexpected, as the tolerance levels from the manufacturers for HVL measurements are greater than or equal to the tolerance levels of the AKR measurements. All measurements were within these bounds of the reference standard HVL, other than the August 2022 (month 0) UW 60‐M measurement with the AGMS‐DM+, with the greatest percent difference from the reference value being +7.3%.

The responses of both XMMs in the UW MO beams were consistent. The Piranha systemically overestimated the HVLs by approximately 5%, and the AGMS‐DM+ systemically underestimated the HVLs by approximately 4%. The energy response of both devices is homogeneous in this low‐energy regime. However, this was not the case for HVL measurements in the UW‐M series beams. The measured HVL for both XMMs increased with respect to the standard value as the tube potential, and therefore, the reference HVL, increased. The one exception was the AGMS‐DM+ measurement of the UW 60‐M beam, where the measured HVL was nearly 4% higher than the reference HVL in month 29 of the study and nearly 6% higher than the reference HVL at the beginning of the study. Again, this is likely the result of the low total filtration used to attenuate that beam.

As seen in Figures 7 and 9, we were unable to acquire an HVL measurement for the UW 150‐M beam with the RTI Piranha. The reference HVL for that beam is toward the high end of the measurable HVLs for the Piranha. The combination of the high tube potential and high total filtration resulted in the HVL being unmeasurable.^15^

### 4.3 Longitudinal study

This nearly 2.5‐year study of XMM performance in the calibration beams has revealed that both devices are relatively consistent beyond their manufacturer‐suggested calibration dates. Given a specific beam and XMM, for any time point in the study, the measured percent difference for the AKR from the reference AKR was within 2% of the initial measured percent difference.

Overall, the Radcal AGMS‐DM+ was the more stable XMM, with only measurements of 2 beams out of the 10 investigated showing statistically significant drift at the p < 0.05 level. This was unexpected, as the AGMS‐DM+ has the shorter suggested calibration period at 1 year, while the RTI Piranha has a 2‐year suggested calibration period. Half of the beam measurements collected with the Piranha showed statistically significant drift, including nearly all of the measurements of the UW‐MO series beams. However, all beam measurements remained within the manufacturers’ specified tolerance levels of the reference standard over the entire course of the study. Additionally, both XMMs satisfied the IEC 61674 energy‐response variation criterion throughout the duration of the study.^3^ Therefore, while the drifts may be statistically significant, their magnitudes are small, given the duration of the study and the manufacturer‐stated uncertainties of the AKR measurements. It must also be noted that only one unit of each XMM model was tested in this study. The results of the longitudinal study are assumed to be representative of all units of each XMM model.

The cause of the drift in the measurements is unclear. The response of a given diode is particularly sensitive to differences in photon energy.^16^ Therefore, changes in the x‐ray beam spectra or sensitivity of the diodes inside each XMM may result in changes in measurement results. The drifts observed in this study may worsen with increased lifetime doses. Finally, the XMMs investigated in this project were not used in a clinical setting, so conclusions on how XMM performance may change over time in clinical x‐ray beams cannot be made.

## 5 CONCLUSIONS

The performance of an RTI Piranha and Radcal Accu‐Gold+ with AGMS‐DM+ sensor was evaluated in low‐energy x‐ray calibration beams. AKR and HVL measurements with both devices were accurate within manufacturer‐specified tolerances. For specific beam series, an additional calibration factor could be applied to the measurement results to improve accuracy further. The stability of the devices’ calibrations over time was examined through repeated acquisitions over the span of nearly 30 months. The AGMS‐DM+ had generally more consistent results compared to the RTI Piranha, but both devices remained within the expected range of the reference AKRs. It is unclear whether using these XMMs in a clinical setting, where the frequency of usage or accumulated lifetime dose may be higher, would have a greater impact on the performance of these devices over time.

## CONFLICT OF INTEREST STATEMENT

The authors have no relevant conflicts of interest to disclose.
